# Antimicrobial Resistance Genes (ARGs) Monitoring and Gut Microbiota Profiling in Honey Bees from an Intensive Livestock Farming Area in Northwestern Italy

**DOI:** 10.3390/microorganisms14050967

**Published:** 2026-04-25

**Authors:** Silvia Olivieri, Roberto Zoccola, Chiara Beltramo, Cecilia Guasco, Luca Carisio, Andrea Trossi, Alessandro Dondo, Simone Peletto, Maria Goria

**Affiliations:** Istituto Zooprofilattico Sperimentale del Piemonte, Liguria e Valle D’Aosta, Via Bologna 148, 10154 Torino, Italy; silvia.olivieri@izsplv.it (S.O.); roberto.zoccola@izsplv.it (R.Z.); chiara.beltramo@izsplv.it (C.B.); cecilia.guasco@izsplv.it (C.G.); luca.carisio@izsplv.it (L.C.); andrea.trossi@izsplv.it (A.T.); alessandro.dondo@izsplv.it (A.D.); maria.goria@izsplv.it (M.G.)

**Keywords:** antimicrobial resistance (AMR), antibiotic resistance genes (ARGs), honey bees, gut microbiota, intensive livestock farming, environmental bioindicators

## Abstract

Antimicrobial resistance (AMR) is a growing global concern, exacerbated by the overuse of antibiotics in livestock farming. Honey bees (*Apis mellifera*), widely used as bioindicators of environmental contamination, may also serve as sentinels for monitoring the environmental spread of antibiotic resistance genes (ARGs). This study investigated the presence of ARGs and the gut microbiota composition of honey bees sampled from 11 apiaries located in a region of Northwestern Italy characterized by intensive livestock farming. PCR and Sanger sequencing analyses revealed a widespread presence of tetracycline resistance genes—particularly *tetB* and *tetC*—as well as occasional detection of *blaTEM*, *qnrB*, and *int1* genes. *tetB* and *tetC* were also identified in three bacterial colonies isolated from bee guts, notably in *Hafnia* spp. 16S rRNA gene sequencing of the gut microbiota revealed dominance of genera such as *Bartonella*, *Snodgrassella*, *Gilliamella*, *Bombilactobacillus*, and *Lactobacillus*. Some samples showed shifts in the microbial diversity. The findings confirm the potential of honey bees as bioindicators for environmental AMR surveillance and underscore the need for further research to elucidate correlations between ARG presence and microbial community structure in honey bees from various ecological contexts.

## 1. Introduction

Antimicrobial resistance (AMR) is a natural evolutionary phenomenon, but its global increase is strongly driven by the widespread and excessive use of antibiotics [1]. Data from the European Centre for Disease Prevention and Control (ECDC) indicate that every year, in Europe alone, 35,000 people die from infections caused by antibiotic resistance, of which 10,000 occur in Italy [2]. According to the ISTISAN Reports 21/3, AMR threatens food security, international trade, and economic development and undermines progress toward the Sustainable Development Goals (SDGs) defined by the World Health Organization (WHO). It also leads to increased healthcare costs associated with prolonged hospital stays, the need for often more expensive therapies, greater use of diagnostic techniques, complications, deaths, and litigation [3].

This problem does not concern only human health but also animal health and, more broadly, the environment. Indeed, antibiotics are among the most abundant compounds found in urban wastewater and surface waters [4].

The selective pressure induced by antibiotic use, and their subsequent release into the environment, has contributed to the mobilization and horizontal gene transfer (HGT) of numerous antibiotic resistance genes (ARGs) among bacterial species [5]. Indeed, ARGs are often carried on mobile genetic elements, such as plasmids, transposons, and integrons, that can transfer genes between bacteria, both intra- and interspecifically [6], making AMR particularly challenging to control.

HGT phenomenon can occur in different environments but particularly when bacterial loads are high, such as in soil, in wastewater treatment plants, and in the gut micro-biome of humans and animals [7]. In recent years, several studies have detected ARGs in wildlife and natural environments [8,9,10], highlighting the importance of monitoring organisms that may be exposed to antibiotics indirectly through environmental contamination. Indeed, even if wildlife is rarely directly exposed to antibiotics in the environment, sharing of common habitats, water sources, and environmental contamination, could play a pivotal role in transfer of ARGs between wildlife and food animals, often directly treat with antibiotics [11].

Honey bees (*Apis mellifera*) have been used for over 50 years as bioindicators of environmental pollution, including heavy metals in urban areas, pesticides in agricultural zones, and radionuclides [12]. Bees forage within a range of up to 3 km from their hives, making them highly exposed to environmental contaminants during contact with soil, air, water, and vegetation [13]. They also have morphological characteristics that make them a good environmental indicator, such as the presence of bristles and hairs on the body surface, useful to capture different types of particles, including contaminants [14]. Furthermore, beekeeping practices allow monitoring of honey bee foraging near their hives, thereby enabling environmental surveillance of a specific area through hive placement. Consequently, honey bees play a unique role, straddling the boundary between managed and wild species, which enhances their potential as effective bioindicators.

Although the use of antibiotics is prohibited in beekeeping within the European Union, bees may still come into contact with these compounds in the environment. Given their ecological characteristics discussed above, honey bees may serve as indicators of the indirect environmental effects of antimicrobial use in healthcare [13,14,15,16,17]. In particular, they can acquire ARGs through bacteria ingested during their foraging activities, for example, from stagnant water, potentially contaminated by several bacteria coming also from human and animals’ feces [18]. Moreover, the detected positive associations between ARG abundance in beehive products and anthropogenic environments suggests that ARGs might originate from the honey bee foraging environment [19].

The honey bee gut microbiota—characterized by high bacterial density like that of other animals—could be a hotspot for ARGs.

The honey bee gut microbiota is highly specialized and conserved, comprising five to nine bacterial species clusters, in which five of these represent the core gut microbiome and can be found in every adult bee worker (*Snodgrassella alvi*, *Gilliamella apicola*, two species of *Lactobacillus*, and a *Bifidobacterium* species); instead, the others are often present in guts of many honey bee workers but sometimes absent (within these, *Bartonella apis* and *Frischella perrara* are widely identified) [20,21,22,23]. In adult worker bees, these bacteria are distributed across specific gut compartments, playing essential roles in nutrient digestion and defense against pathogens [21].

However, the relative abundance of these bacteria can fluctuate seasonally, depending on the diversity and availability of food sources [24].

Disruption of the gut microbial community—often marked by the emergence of opportunistic bacteria such as Enterobacteriaceae—can occur due to external stressors, including antibiotic exposure [25]. Several studies have linked antibiotic exposure to dysbiosis in bees, leading to increased vulnerability to pathogens, such as *Nosema ceranae*, honey bee viruses and *Melissococcus plutoniae* [26,27,28,29,30]. The administration of antibiotics such as tylosin, sulfonamides, and tetracycline under experimental conditions can influence the gut microbiota composition in bees, changing the ratios among the taxa [27].

At the global scale, the intensive use of antibiotics in livestock has been one of the most important factors driving AMR [31,32]. Recently, the European Union has opted for a more parsimonious use of antibiotics in livestock, aiming to prevent the occurrence and spread of ARGs [33]. In Italy, the strategy to reduce antibiotic use in livestock is supported by an information system [34], which collects data on drug use at the farm level. Surveillance of antibiotic use will be enhanced by the data collected through ClassyFarm; however, at present, these data are still limited. Meanwhile, the intensification of environmental surveillance methods—such as those provided by honey bees—remain a key tool to monitor the spread and occurrence of antibiotic resistance genes.

In this study, we conducted a surveillance across apiaries located in an area characterized by intensive livestock farming. The aim was to investigate the presence of ARGs in the honey bee gut and to collect data on the gut microbial community composition. The selected area may represent a potential hotspot for ARGs and environmental antibiotic contamination due to the high density of livestock farming. Therefore, we analyzed both the occurrence of ARGs and the composition of gut microbial community in *A. mellifera* collected from different apiaries within this area. Given the limited knowledge on the spread of ARGs, particularly between clinical context and the natural environment, this study aimed to evaluate the potential role of honey bees as indicators of environmental ARG contamination and to analyze the gut microbial community structure.

## 2. Materials and Methods

### 2.1. Study Area and Sampling

**Figure 1:**
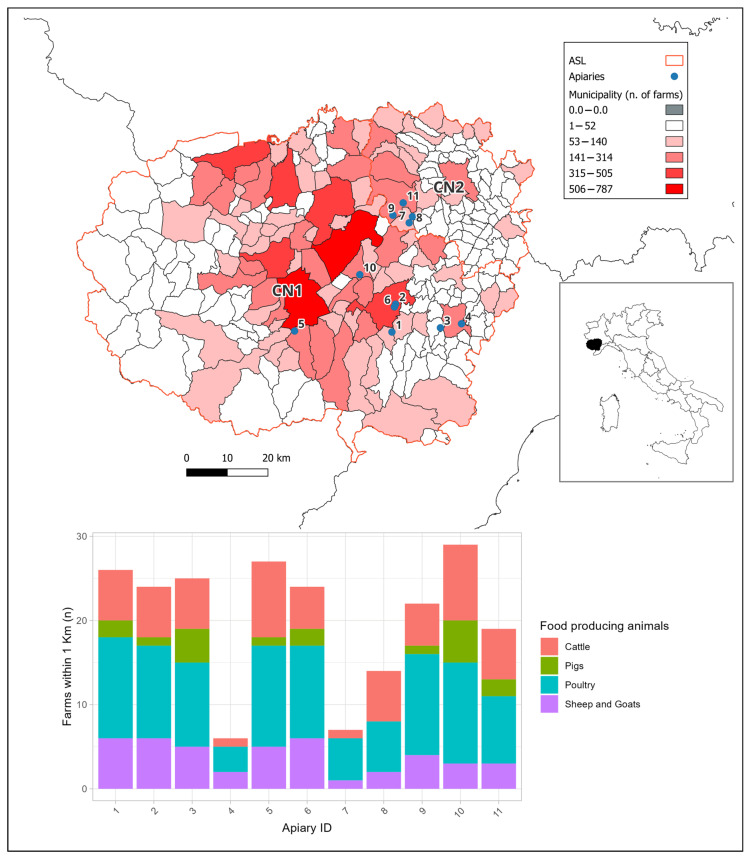
Apiary locations and number of livestock farms in the surrounding area. The map shows the study area in the Piedmont region (northwestern Italy), including the local health authority units (ASL) CN1 and CN2. In the map, the red scale indicates the number of livestock farms within each municipality. The bar plot shows the number of farms located within a 1 km radius of the sampled apiaries.

Sampling was carried out during the spring and summer seasons of 2021 and 2022, across 11 apiaries located in the Province of Cuneo, Piedmont (Northwestern Italy), an area characterized by a high density of intensive livestock farms (Figure 1). As of 31 December 2021, the density of farm animals in the area was 895 animals/km^2^ for poultry, 131 for pigs, 62 for cattle, and 8 for sheep or goats. This area is recognized as a hotspot for dairy, beef, and pig farming at the national level in Italy, and it has an intermediate livestock density compared with other European regions [35].

As illustrated in Figure 1, numerous farms housing various food-producing animals are situated in close proximity to the sampled apiaries.

As detailed in Table 1, three hives were sampled at each apiary and four aliquots with about 30 bees were collected for each hive. The bees were collected from the hive using a perforated plastic container. They were then transported to the reference laboratory and stored at −20 °C. For each Apiary ID and Colony ID, sampling was carried out only one time. Of the four aliquots, one was used for ARGs determination in isolated colonies, one was used for ARG determination in gut and for microbiota analysis (using the same extracted DNA), and the remaining two were stored for other future analysis.

The beekeepers were interviewed about the technical and sanitary management of the apiaries. Overall, the apiaries were in good condition, with all colonies having an active queen and more than 60% of colony frames populated by bees. However, colonies in Apiaries 5 and 6 were noted to be in poor health due to past depopulation, mortality, robbing, and severe losses caused by *Varroa* infestation.

### 2.2. ARG Determination in Honey Bee Gut

For the determination of antimicrobial resistance genes (ARGs), one aliquot from each sample was used with a total of 30 individuals.

The target genes were selected based on the main use of the antibiotics to which they are resistant in veterinary clinical settings and consulting the WHOA List of Antimicrobial Agents of Veterinary Importance [36]. We also considered the findings available in the literature regarding AMR and honey bees. Overall, the selection of *tetA*, *tetB*, *tetC*, *tetD*, *tetE*, *tetG*, *qnrA*, *qnrB*, *qnrS*, *gyrA*, *blaTEM*, *intl1*, and *intl2* was based on their high epidemiological relevance; widespread occurrence across environmental-, animal-, and human-associated microbiomes; and their frequent association with mobile genetic elements.

The honey bee gut was carefully separated from the rest of the body using sterile tweezers. DNA extraction was performed with the Qiamp DNA Mini Kit (Qiagen, Hilden, Germany) following the manual instructions. DNA extracted was quantified, setting a spectrophotometer at 260 nm (BioSpectrometer^®^ (Invitrogen, Thermo Fisher Scientific, Waltham, MA, USA).

The selected ARGs are listed in Table 2. The target genes were chosen based on their association with resistance to the following antibiotics classes: tetracyclines, cephalosporins, and fluoroquinolones. Additionally, integrase genes were detected to investigate the phenomenon of multidrug resistance. Simplex PCR end point was performed in a final volume of 25 µL, using 0.5 µM of each primer, 2.5 U of Taq HOT Start type, 2 mM of MgCl2, 0.3 mM of DNTPs mix, and 5 µL of DNA sample.

Primers and thermal profile for detecting the genes responsible for cephalosporin resistance were taken from Nagano et al., 2003 [37]. However, primers and thermal profile for amplifying the genes responsible for tetracycline resistance were taken from Ng et al., 2001 [38]. Finally, primers and thermal profile used to amplify the genes responsible for fluoroquinolone resistance were taken from Gay et al., 2006 [39]. For each ARG target, positive amplification controls identified in previous studies were included, and as negative control, deionized water was used.

The PCR reaction was carried out using a Gene Amp PCR System 9700 (Applied Biosystems, Foster City, CA, USA). Thermal cycling conditions are shown in Table 2. The PCR products were analyzed by electrophoresis on a 2% Agarose gel, and amplicons corresponding to the expected sizes of the ARGs were sequenced. PCR products were purified using the Extractme DNA Clean-Up Kit (Blirt SA, Blirt, Danzig, Poland). Sequencing was carried out using the BigDye Terminator v3.1 kit (Thermo Fisher Scientific, Waltham, MA, USA ), with the same primers used for PCR. The sequencing products were purified with DyeEX 2.0 Spin Kit (Qiagen, Hilden, Germany), and capillary electrophoresis was performed using the 3500 Series Genetic Analyzer (Thermo Fisher Scientific, Waltham, MA, USA). The forward and reverse sequences obtained were verified and aligned using BioEdit software version 7.7.1 (2021), and a consensus sequence was generated. The consensus sequence was uploaded to the GenBank platform via the Standard Nucleotide Blast interface and compared with the sequences in the database using the Megablast function.

### 2.3. ARG Determination in Isolated Colonies

One of the four aliquots for each sample was used for the phenotypical analysis, starting with the separation of the gut. For each apiary, 30 separated guts were homogenated, and to enrich the sample, 500 µL of each homogenate was mixed with 4.5 mL of Nutrient Broth and incubated over night at room temperature. The culture was then inoculated onto two blood agar plates and incubated at room temperature under aerobic conditions. After 24 h, the plates were examined, and if no growth was observed, the incubation period was extended for up to 72 h.

Single, isolated colonies were picked with a loop and diluted in 500 µL of ultrapure water. DNA extraction was performed by thermal lysis (boiling) of the diluted colonies. The DNA obtained in this way was used to identify bacterial species by amplifying a 530-bp region of the 16S rDNA gene, using the commercial MicroSEQ™ 500 16S rDNA Sequencing kit (Thermo Fisher Scientific, Waltham, MA USA) according to the manufacturer’s instructions. Then, 15 µL of DNA was added to 15 µL of PCR Master MIX and subjected to 30 amplification cycles (95 °C for 30 s, 60 °C for 30 s, and 72 °C for 45 s). The PCR products were analyzed by electrophoresis on a 2% agarose gel and purified using the Extractme DNA Clean-Up Kit (Blirt SA, Danzig, Poland). The purified products were subjected to Sanger sequencing according to the kit instructions (Forward-sequencing reaction—Combine 7 µL of purified PCR product with 13 µL of forward sequence mix; Reverse-sequencing reaction—Combine 7 µL of purified PCR product with 13 µL of reverse sequence mix).

The sequencing products were purified using the DyeEX 2.0 Spin Kit (Qiagen, Hilden, Germany) and analyzed as described in the previous paragraph. Species-level identification was accepted only when sequence similarity met the thresholds recommended by the MicroSEQ database; otherwise, identification was reported at the genus level.

### 2.4. Evaluation of Gut Microbial Community Composition

DNA extracted for ARG determination was quantified with Qubit dsDNA HS Kit (ThermoFisher) and normalized to 5 ng/µL for the 16S rRNA metabarcoding. This is a powerful NGS technique used to identify and quantify bacterial communities in samples associated with environmental conditions or hosts by targeting the hypervariable regions V3 and V4 of the 16S gene. Usually, taxonomical identification is limited to the genus or family level due to intrinsic limitation of the analysis, such as the sequence length or the accuracy of reference databases. The protocol suggested by Illumina (16S Metagenomic Sequencing Library Preparation) was followed for 16S ribosomal metabarcoding. The V3-V4 region was amplified with the primers 341F and 806R [40,41], with an adapter added for the attachment of Illumina indexes.

A no-template control was included, processed alongside the samples to detect potential contamination. The Amplicon PCR reaction was prepared using 12.5 µL of NEBNext^®^ Q5^®^ Hot Start HiFi 2X Master Mix (New England Biolabs, Ipswich, MA, USA), 1.25 µL of 10 µM Forward Primer, 1.25 µL of 10 µM Reverse Primer, 7.5 µL of H2O, and 2.5 µL of DNA (5 ng/µL). The thermal cycling conditions were as follows: 98 °C for 30s; 35 cycles of 98 °C for 10s; 55 °C for 30s; 72 °C for 30s; final extension at 72 °C for 2 min. PCR products were analyzed by electrophoretic run on 2% Agarose gel. The amplicons were purified using magnetic beads (AMPure XP, Beckman Coulter, Brea, CA, USA) directly on a 96-well plate.

The Index PCR was performed using 25 µL of NEBNext^®^ Q5^®^ Hot Start HiFi 2X Master Mix (New England Biolabs, Ipswich, MA, USA), 5 µL of Nextera XT Index Primer 1, 5 µL of Nextera XT Index Primer 2 (Illumina), 10 µL of H2O, and 5 µL of purified DNA with the following thermal conditions: 98 °C for 30s; 12 cycles of 98 °C for 10s; 55 °C for 30s; 72 °C for 30s; final extension at 72 °C for 2 min.

After purification with magnetic beads (AMPure XP, Beckman Coulter, Brea, CA, USA), the libraries were quantified using the Qubit dsDNA HS (ThermoFisher, Waltham, MA USA, and the average size of the amplicons was evaluated using the BioAnalyzer 2100 with the Agilent High Sensitivity DNA Kit (Agilent Technologies, Santa Clara, CA, USA)). The libraries were then normalized and pooled together. The pool was quantified using the NEBNext Library Quant Kit for Illumina (New England Biolabs, Ipswich, MA, USA), normalized to 4 nM, and subjected to paired-end 2x300 sequencing on an Illumina MiSeq platform using the MiSeq Reagent Kit v3 (600-cycle) (Illumina, San Diego, CA, USA).

### 2.5. Data Processing and Analysis

The raw Fastq data obtained from 16S metabarcoding were analyzed using the Microbial Genomics Module in CLC Genomics Workbench v. 23.0.2 (Qiagen). The paired end reads data were jointed and processed using the “Data QC and OTUs Clustering” and “Estimate Alpha and Beta Diversity” workflow tools. Specifically, trimming was performed for low-quality scores (Qscore < 0.05), nucleotide ambiguity (up to 2 nucleotides allowed), adapter sequences, and length. Chimeric reads were removed, duplicate sequences were merged, and the resulting reads were aligned against the SILVA database (v. 138) at a 97% identity threshold, after filtering and removing the accessions with a description related to “Mitochondria” and “Chloroplast”. An operational taxonomic units (OTU) table was then created, showing the assigned taxonomy. The profiles of the positive and negative controls were verified to exclude cross-contamination and subsequently removed. The samples were compared at a sequencing depth of 3000 reads. Alpha diversity (diversity within the groups) was estimated using total number, Chao-1 bias-corrected, Simpson’s index, and Shannon entropy, while the Bray–Curtis method and principal coordinates analysis (PCoA) were used for the estimation of the beta diversity (diversity between groups).

Confidence intervals for the percentages of positive samples to ARGs and ARG co-occurrence were calculated using the Pearson–Klopper exact method.

## 3. Results

### 3.1. Prevalence of ARGs in Honey Bee Gut

The results of PCR and Sanger sequencing revealed a widespread presence of antibiotic resistance in the honey bee gut, particularly to tetracycline.

The *tetB* target gene was detected in all sample (33), but its presence was not confirmed by sequencing in three samples due to inconsistent fragment intensities. On the other hand, the *tetC* target gene was found in 18 samples. The *blaTEM* gene was detected and confirmed by sequencing in 14 samples. Resistance to fluoroquinolones was also observed in 14 samples, specifically with the *qnrB* target gene, although this finding was not confirmed by sequencing.

Additionally, the *intl1* gene cassette, associated with multidrug resistance, was detected in one out of 33 samples. No positivity was observed for the other tested markers. A summary of the identified markers is provided in Table 3, in which only detected genes are shown. The percentage of positive samples and their relative confidence intervals are indicated in Table 4, and the analysis of co-occurrence of genes is shown in Table 5. All data are presented both as PCR results and as PCR results confirmed by Sanger sequencing.

### 3.2. Colony Identification and ARGs

A total of 20 pure colonies were isolated, 17 of which were identified through 16S rDNA region sequencing as belonging to nine genera: *Enterococcus* (five colonies), *Hafnia* (three colonies), *Bacillus* (two colonies), *Pantoea* (two colonies), *Metabacillus* (one colony), *Paenibacillus* (one colony), *Fructobacillus* (one colony), *Enterobacter* (one colony), and *Staphylococcus* (one colony). Antimicrobial resistance screening performed on individual colonies revealed the presence of tetracycline resistance genes in three colonies, all identified as *Hafnia* and originating from the same apiary and colony (sample 11A). In two of these *Hafnia* colonies, both *tetB* and *tetC* resistance genes were detected, while the third colony only exhibited resistance to *tetC.*

### 3.3. Gut Microbial Community Composition

The FASTQ files (BioProject ID: PRJNA1439716) generated by NGS sequencing on the Illumina MiSeq platform were imported into CLC Genomics Workbench v. 23 (Qiagen) for subsequent bioinformatic analysis (Supplementary Material Table S1). After filtering and trimming, a total of 1012.024 reads were obtained. All samples were deemed suitable for analysis, except for sample 7A, which was excluded from further processing due to insufficient quality (i.e., low read number).

The sequences were clustered into 66 operational taxonomic units (OTUs). The positive control yielded results consistent with expectations, while the negative control and extraction blank showed no signs of contamination.

Analysis of the sequences obtained from wild and farmed samples revealed that the most abundant phyla were *Proteobacteria*, *Firmicutes*, and *Actinobacteria*.

At the class level, *Alphaproteobacteria*, *Gammaproteobacteria*, *Bacilli*, *Actinobacteria*, and *Clostridia* were the most represented, accounting for over 98% of the microbiota.

**Figure 2:**
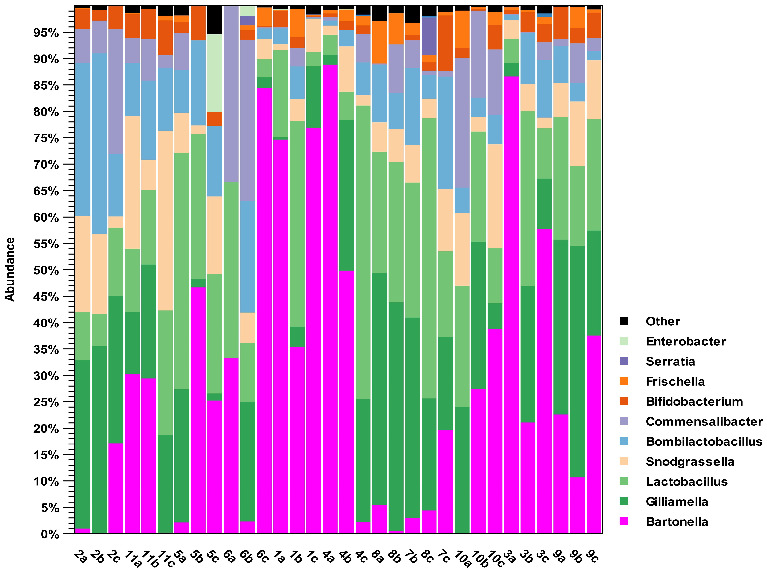
Relative abundance of bacterial communities at the genus level for each individual sample. The legend shows the 15 most abundant genera.

Figure 2 illustrates the taxonomic composition of the microbiota at the genus level for each sample. The five most abundant genera were *Bartonella*, *Gilliamella*, *Lactobacillus*, *Snodgrassella*, *Bombilactobacillus*, and *Commensalibacter.*

Notably, the presence of *Bartonella* exhibited highly variable among samples, with proportions ranging from 0% to over 85% of the microbiota.

The other genera were present at lower abundance (Supplementary Material Table S2): among them there are *Bifidobacterium*, *Frischella*, *Serratia*, and *Enterobacter*.

**Figure 3:**
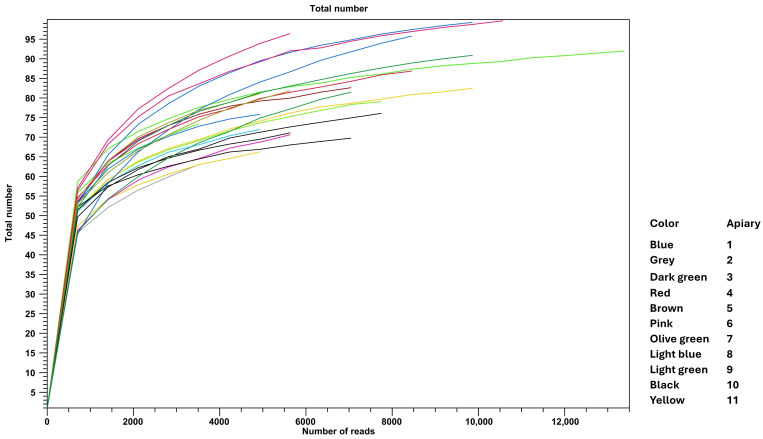
Alpha diversity rarefaction curves of the microbial richness of the samples grouped for apiary.

Considering alpha diversity, the observed number of species ranged from 60.59 to 82.50 (Figure 3), while the Chao1 richness estimator ranged from 71.15 to 87.49. Higher values were observed for samples 1c, 4a, 1a, and 4b, with Chao1 estimates of 92.67, 96.24, 98.49, and 102.15, respectively.

The Simpson index ranged from 0.91 to 0.96, with lower values recorded for samples 4a (0.60), 3a (0.67), 6c (0.68), and 1c (0.75). Similarly, the Shannon index ranged from 4.33 to 5.04, except for samples 4a (2.90), 1c (2.96), 6c (3.08), and 3a (3.20).

**Figure 4:**
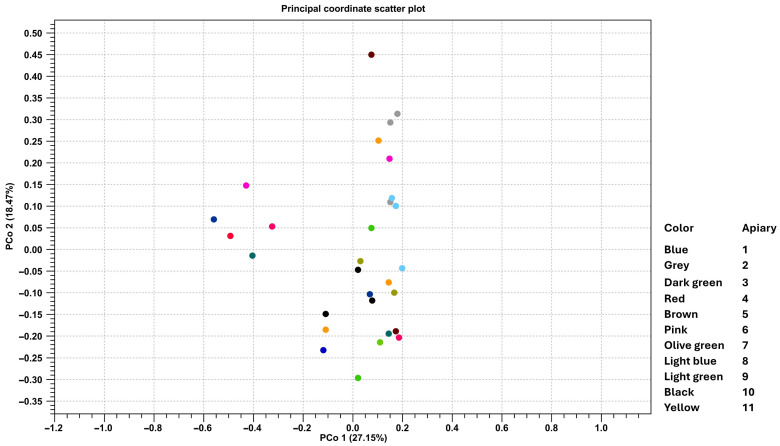
Beta diversity shown by principal coordinates analysis (PCoA) of Bray–Curtis method. The samples are grouped for apiary.

Beta diversity analysis (Figure 4) showed that samples were dispersed across the ordination space without forming distinct clusters.

## 4. Discussion

The presence of antibiotic resistance genes (ARGs) in the natural environment is now widely recognized, emphasizing the urgency of addressing the antimicrobial resistance (AMR) emergency through a One Health approach. All environmental matrices—including water [42,43], soil [44,45], and air [46,47,48]—can serve as sources of ARGs. Although ARGs have been detected even in remote areas [49,50], the presence of urban centers and livestock or agricultural farms can create hotspots that intensify the AMR phenomenon [51].

Honey bees have emerged as valuable bioindicators for monitoring the environmental presence of ARGs in both natural and anthropogenic contexts, particularly due to the strict regulations on antibiotic use in EU beekeeping that forbid a direct exposure to these drugs for clinical purposes [19]. Their foraging behavior makes them suitable for localized environmental monitoring as they typically forage within a 3 km radius from their hive, during which they collect a variety of environmental materials, including potential contaminants.

Various environmental variables are known to promote the spread of ARGs. Recent studies [13,16] have identified correlations between the AMR phenomenon in honey bees and specific landscape features, such as the proximity to livestock farms, agricultural areas, wastewater treatment plants, and hospitals.

Furthermore, analyzing the microbial community composition of honey bees may play an important role in detecting dysbiosis associated with bees exposed to various stressors, compared to healthy individuals [20,25,27,52]. This is particularly relevant for the diagnosis and research of disease symptoms in honey bees, which may originate from diverse causes, ranging from pathogens to xenobiotics. Shifts in the microbial community structure have also been linked to territorial and environmental characteristics [53,54].

In this study, the sampling area was characterized by a high density of intensive livestock farms. Approximately 10% of the national livestock population, corresponding to about 1 million livestock units (LUs), is reared in the Piedmont region. Within the region, the Province of Cuneo accounts for the highest concentration of livestock, representing the largest zootechnical area in Piedmont.

All sampled apiaries were in close proximity to different livestock farms and, except for apiaries 4, 7, and 8, there were more than 15 farms within 1 km radius from the apiaries. These farms rear various species, including cattle, poultry, pigs, sheep, and goats. This homogeneity within the sampling area leads to expected results that do not differ significantly; however, it remains useful for collecting information on ARG presence in the area and variability in gut microbial community composition.

The results revealed a widespread presence of tetracycline resistance genes, according to previous studies [14,55]. The investigated *tet* genes are detected both in honey bee gut and in isolated colonies of *Hafnia*. The global prevalence of tetracycline resistance genes could be attributed to the historic use of tetracyclines in beekeeping prior to the implementation of EU regulations [17]. Moreover, tetracycline is one of the most commonly used antimicrobials in veterinary clinical practice [56]; therefore, these findings further support the role of tetracycline resistance genes as proxies for environmental antimicrobial resistance pollution The detection of *blaTEM* in a subset of samples further indicates the occurrence of β-lactam resistance determinants within the bee-associated microbiota. Given that *blaTEM* is frequently associated with mobile genetic elements, its presence may reflect environmental dissemination driven by the extensive use of β-lactam antibiotics, including penicillins and cephalosporins, in both human and veterinary medicine. Overall, the co-occurrence analysis of genes reveals a widespread presence of resistance in the analyzed samples, with 97% of samples harboring at least one resistance gene, based on PCR results confirmed by Sanger sequencing.

This finding supports the hypothesis that honey bees can be exposed to, and potentially contribute to, the circulation of clinically relevant resistance genes in the environment. Gut microbiota analysis showed that the genus *Bartonella* was the most abundant across samples, followed by *Snodgrassella*, *Gilliamella*, *Bombilactobacillus*, and *Lactobacillus. Bartonella* spp. were detected in all apiaries except one. Similarly, *Gilliamella*, *Bombilactobacillus*, and *Snodgrassella* were also present in all but one apiary. In contrast, *Lactobacillus* was found in all colonies with varying abundance. These results confirmed the presence of the key bacterial taxa usually present in the gut microbiota of bees, which are *Gilliamella*, *Snodgrassella apis*, and *Lactobacillus*. These genera play an important role in host physiology. In particular, *Gilliamella* helps in degradation of pollen pectin and production of antimicrobial peptides; *Snodgrassella apis* contributes to regulation of antimicrobial peptide expression; *Lactobacillus* plays a crucial role in carbohydrate metabolism and contributes to host defense through the production of antimicrobial metabolites and sugars [57]. The presence of a relatively conserved microbiota in the gut of the analyzed samples was suggested also by the alpha and beta diversity analysis: the Chao 1 bias corrected index value near 100 and the high values of Simpson’s index and Shannon entropy showed a relatively simple and homogeneous microbial community, without highlighting significative grouping among apiaries or hives.

Other important genera were *Frischella* and *Bartonella*, often identified in the gut bacterial community but with less abundance and low prevalence [21]. In the analyzed samples, while *Bartonella* was very abundant, *Frischella* was absent in some samples, such as 2B, 2C, 5B, 5C, 6A, 9A, and 11B.

Notably, samples 2A and 2B exhibited reduced abundance of *Bartonella*, accompanied by an increased presence of *Bombilactobacillus*. According to previous research [17], a decline in *Bartonella* spp. abundance may be linked to compromised health status. That study found *Bartonella apis* to be relatively more abundant in healthy bees compared to bees from collapsing colonies, suggesting a potential role in disease resistance. *Bartonella* spp. are known to play a role in recycling nitrogenous waste into amino acids and in the degradation of secondary plant metabolites; therefore, a reduction of over 80% in this genus, as observed in some samples, could have implications for digestion and amino acid recovery [17].

It is also noteworthy that sample 8A showed 4.6% of OTUs belonging to the genus *Serratia*. It can act as a pathogen, and its presence is particularly significant in bees that may already be experiencing compromised health conditions. Perturbation of the gut microbiota can promote lethal infection, causing great mortality rate in bees [58].

## 5. Conclusions

This study confirms that honey bees could come into contact with ARGs, supporting their potential use as bioindicators for monitoring the presence of these environmental contaminants in an area characterized by intensive livestock farming. Nevertheless, more data and studies are necessary to understand whether the presence of farming activities or other potential hot spots for ARGs could influence honey bee exposure to these contaminants. It would also be valuable to clearly identify the most representative target genes across different environmental contexts.

The analysis of gut microbial community composition may serve as a valuable indicator for honey bee health status in potentially contaminated areas. However, numerous environmental and biological variables influence the composition of the gut microbiota, often complicating the interpretation of results.

Further research, involving a larger number of samples and comparisons between bees from different environmental contexts, is needed to better understand this complex phenomenon and to clarify whether the presence of ARGs can be reliably correlated with shifts in microbial community composition in this territorial context.

This work confirms the importance of monitoring studies to support surveillance of AMR and its environmental dissemination.

## Figures and Tables

**Table 1:** Sampling number and apiary localization.

Apiary ID	Colony ID	Sampling Date	Municipality	Coordinate WGS 84
1	A	27-07-2022	Monastero di Vasco (CN)	44.348637 (N) 7.832953 (E)
	B			
	C			
2	A	15-09-2021	Mondovì (CN)	44.410084 (N) 7.843101 (E)
	B			
	C			
3	A	27-07-2022	Monbasiglio (CN)	44.359357 (N) 7.982282 (E)
	B			
	C			
4	A	27-07-2022	Malpotremo (CN)	44.368907 (N) 8.047212 (E)
	B			
	C			
5	A	26-07-2022	Boves (CN)	44.347433 (N) 7.532728 (E)
	B			
	C			
6	A	27-07-2022	Mondovì (CN)	44.404124 (N) 7.839416 (E)
	B			
	C			
7	A	05-09-2022	Narzole (CN)	44.589716 (N) 7.881601 (E)
	B			
	C			
8	A	05-09-2022	Narzole (CN)	44.603442 (N) 7.891445 (E)
	B			
	C			
9	A	26-05-2021	Cherasco (CN)	44.605938 (N) 7.830899 (E)
	B			
	C			
10	A	04-09-2022	Sant’Albano Stura (CN)	44.473888 (N) 7.731156 (E)
	B			
	C			
11	A	15-09-2021	Cherasco (CN)	44.633413 (N) 7.862137 (E)
	B			
	C			

**Table 2:** List of ARGs with their Nucleotide sequences and thermal cycling details.

Target Gene	Antibiotics	Nucleotide Sequence 5′-3′	AMPL (bp)	Amplification
*tetA*	Tetracyclines	GCTACATCCTGCTTGCCTTC CATAGATCGCCGTGAAGAGG	210	94 °C × 60′′, 55 °C × 60″, 72 °C × 90″, 72 °C × 7′ × 35 cycles
*tetB*	Tetracyclines	TTGGTTAGGGGCAAGTTTTG GTAATGGGCCAATAACACCG	659	94 °C × 60′′, 55 °C × 60″, 72 °C × 90″, 72 °C × 7′ × 35 cycles
*tetC*	Tetracyclines	CTTGAGAGCCTTCAACCCAG ATGGTCGTCATCTACCTGCC	418	94 °C × 60′′, 55 °C × 60″, 72 °C × 90″, 72 °C × 7′× 35 cycles
*tetD*	Tetracyclines	AAACCATTACGGCATTCTGC GACCGGATACACCATCCATC	787	94 °C × 60′′, 55,5 °C × 60″, 72 °C × 90″, 72 °C × 7′ × 35 cycles
*tetE*	Tetracyclines	AAACCACATCCTCCATACGC AAATAGGCCACAACCGTCAG	278	94 °C × 60′′, 55 °C × 60″, 72 °C × 90″, 72 °C × 7′ × 30 cycles
*tetG*	Tetracyclines	GCTCGGTGGTATCTCTGCTC AGCAACAGAATCGGGAACAC	468	94 °C × 60′′, 55 °C × 60″, 72 °C × 90″, 72 °C × 7′ × 35 cycles
*qnrA*	Fluoroquinolones	ATTTCTCACGCCAGGATTTG GATCGGCAAAGGTTAGGTCA	516	94 °C × 45′′, 53 °C × 45″, 72 °C × 60″, 72 °C × 7′ × 32 cycles
*qnrB*	Fluoroquinolones	GATCGTGAAAGCCAGAAAGG ACGATGCCTGGTAGTTGTCC	469	94 °C × 45′′, 53 °C × 45″, 72 °C × 60″, 72 °C × 7′ × 32 cycles
*qnrS*	Fluoroquinolones	ACGACATTCGTCAACTGCAA TAAATTGGCACCCTGTAGGC	417	94 °C × 45′′, 53 °C × 45″, 72 °C × 60″, 72 °C × 7′ × 32 cycles
*gyrA*	Fluoroquinolones	ATGAGCGACCTTGCGAGAGAAATTACACCG TTCCATCAGCCCTTCAATGCTGATGTCTTC	630	94 °C × 30′′, 55 °C × 30″, 72 °C × 30″, 72 °C × 7′ × 30 cycles
*blaTEM*	Cephalosporines	CCGTGTCGCCCTTATTCC AGGCACCTATCTCAGCGA	823	94 °C × 60′′, 55 °C × 60″, 72 °C × 90″, 72 °C × 7′ × 30 cycles
*blaSHV*	Cephalosporines	ATTTGTCGCTTCTTTACTCGC TTATGGCGTTACCTTTGACC	526	94 °C × 60′′, 55 °C × 60″, 72 °C × 90″, 72 °C × 7′ × 30 cycles
*blaCTX-M-1*	Cephalosporines	CGGTGCTGAAGAAAAGTG TACCCAGCGTCAGATTAC	353	94 °C × 60′′, 55 °C × 60″, 72 °C × 90″, 72 °C × 7′ × 30 cycles
*blaCTX-M-2*	Cephalosporines	ACGCTACCCCTGCTATTT CCTTTCCGCCTTCTGCTC	764	94 °C × 60′′, 55 °C × 60″, 72 °C × 90″, 72 °C × 7′ × 30 cycles
*blaCTX-M-9*	Cephalosporines	GCAGATAATACGCAGGTG CGCCGTGGTGGTGTCTCT	392	94 °C × 60′′, 55 °C × 60″, 72 °C × 90″, 72 °C × 7′ × 30 cycles
*intl1*	Multiresistance	GGGTCAAGGATCTGGATTTCG ACATGGGTGTAAATCATCGTC	483	94 °C × 30′′, 62 °C × 30″, 72 °C × 30″, 72 °C × 7′× 30 cycles
*intl2*	Multiresistance	CACGGATATGCGACAAAAAGGT GTAGCAAACGAGTGACGAAATG	788	94 °C × 30′′, 62 °C × 30″, 72 °C × 30″, 72 °C × 7′ × 30 cycles

**Table 3:** Presence of ARGs in honey bee gut samples analyzed with Simplex PCR end point. The symbol + indicates the target genes detected by PCR and confirmed by Sanger sequencing. Not confirmed indicates the target gene detected by PCR but not confirmed by Sanger sequencing due to inconsistent fragment intensities.

Sample ID	*blaTEM*	*tetB*	*tetC*	*qnrB*	*intl1*
1A	-	+	-	-	-
1B	-	+	+	Not confirmed	-
1C	-	+	Not confirmed	Not confirmed	-
2A	+	Not confirmed	+	Not confirmed	-
2B	+	+	+	Not confirmed	-
2C	+	+	+	-	+
3A	+	+	+	Not confirmed	-
3B	-	+	+	-	-
3C	-	+	-	Not confirmed	-
4A	+	+	Not confirmed	-	-
4B	+	+	+	Not confirmed	-
4C	+	+	+	Not confirmed	-
5A	+	+	Not confirmed	Not confirmed	-
5B	+	+	-	Not confirmed	-
5C	-	+	+	Not confirmed	-
6A	-	+	Not confirmed	-	-
6B	-	+	+	-	-
6C	-	+	Not confirmed	-	-
7A	+	+	-	Not confirmed	-
7B	-	+	+	-	-
7C	-	+	+	-	-
8A	-	+	+	-	-
8B	-	+	+	-	-
8C	-	+	+	-	-
9A	+	+	Not confirmed	-	-
9B	-	Not confirmed	-	-	-
9C	+	Not confirmed	+	-	-
10A	-	+	Not confirmed	-	-
10B	-	+	Not confirmed	-	-
10C	-	+	-	Not confirmed	-
11A	+	+	+	-	-
11B	+	+	+	Not confirmed	-
11C	-	+	Not confirmed	-	-

**Table 4:** Proportion of positive samples for each gene with confidence intervals.

Type of Test	Target Gene	Negative	Positive	Result
PCR	*blaTEM*	19	14	42.4% (25.5–60.8)
	*tetB*	0	33	100% (89.4–100)
	*tetC*	6	27	81.8% (64.5–93)
	*qnrB*	19	14	42.4% (25.5–60.8)
	*intl1*	32	1	3% (0.1–15.8)
PCR + Sanger	*blaTEM*	19	14	42.4% (25.5–60.8)
	*tetB*	3	30	90.9% (75.7–98.1)
	*tetC*	15	18	54.5% (36.4–71.9)
	*qnrB*	33	0	0% (0–10.6)
	*intl1*	32	1	3% (0.1–15.8)

**Table 5:** Proportion of gene co-occurrence with confidence intervals.

Type of Test	Number of Co-Occurring Genes	Negative	Positive	Result
PCR	≥1	0	33	100% (89.4–100)
	≥2	2	31	93.9% (79.8–99.3)
	≥3	16	17	51.5% (33.5–69.2)
	≥4	25	8	24.2% (11.1–42.3)
	≥5	33	0	0% (0–10.6)
PCR + Sanger	≥1	1	32	97% (84.2–99.9)
	≥2	10	23	69.7% (51.3–84.4)
	≥3	26	7	21.2% (9–38.9)
	≥4	32	1	3% (0.1–15.8)
	≥5	33	0	0% (0–10.6)

## Data Availability

The data supporting the findings of this study are available within the manuscript. Additional information is available from the corresponding author upon reasonable request.

## Supplementary Materials

The following supporting information can be downloaded at: https://www.mdpi.com/article/10.3390/microorganisms14050967/s1, Table S1: The table represents the initial number of reads and the number of reads after trimming, filtered based on number, filtered based on non-chimeric reads, and classified in OTU table for each sample. Table S2: The OTU table shows for each sample the bacterial genera sequenced and the corresponding number of reads.

## References

[1] OECD (2022). Antimicrobial Resistance—Tackling the Burden in the EU. Organization for Economic Co-Operation and Development.

[2] ECDC Surveillance of Antimicrobial Resistance in Europe, 2022 Data. https://www.europa.eu.

[3] Istituto Superiore di Sanità (2021). Rapporti ISTISAN. Approccio Ambientale All’antimicrobico Resistenza.

[4] Castiglioni S., Davoli E., Riva F., Palmiotto M., Camporini P., Manenti A., Zuccato E. (2018). Mass balance of emerging contaminants in the water cycle of a highly urbanized and industrialized area of Italy. Water Res..

[5] Larsson D.G.J., Flach C.F. (2022). Antibiotic resistance in the environment. Nat. Rev. Microbiol..

[6] Vittecoq M., Godreuil S., Prugnolle F., Durand P., Brazier L., Renaud N., Arnal A., Aberkane S., Jean-Pierre H., Gauthier-Clerc M. (2016). Antimicrobial resistance in wildlife. J. Appl. Ecol..

[7] McInnes R.S., McCallum G.E., Lamberte L.E., van Schaik W. (2020). Horizontal transfer of antibiotic resistance genes in the human gut microbiome. Curr. Opin. Microbiol..

[8] Allen H.K., Donato J., Wang H.H., Cloud-Hansen K.A., Davies J., Handelsman J. (2010). Call of the wild: Antibiotic resistance genes in natural environments. Nat. Rev. Microbiol..

[9] Schwartz T., Kohnen W., Jansen B., Obst U. (2003). Detection of antibiotic-resistant bacteria and their resistance genes in wastewater, surface water, and drinking water biofilms. FEMS Microbiol. Ecol..

[10] Wang W., Weng L., Luo T., Wang Q., Yang G., Jin Y. (2023). Antimicrobial and the Resistances in the Environment: Ecological and Health Risks, Influencing Factors, and Mitigation Strategies. Toxics.

[11] Greig J., Rajić A., Young I., Mascarenhas M., Waddell L., LeJeune J. (2015). A Scoping Review of the Role of Wildlife in the Transmission of Bacterial Pathogens and Antimicrobial Resistance to the Food Chain. Zoonoses Public Health.

[12] Celli G., Maccagnani B. (2003). Honey bees as bioindicators of environmental pollution. Bull. Insectology.

[13] Cenci-Goga B.T., Sechi P., Karama M., Ciavarella R., Pipistrelli M.V., Goretti E., Elia A.C., Gardi T., Pallottini M., Rossi R. (2020). Cross-sectional study to identify risk factors associated with the occurrence of antimicrobial resistance genes in honey bees (*Apis mellifera*) in Umbria, Central Italy. Environ. Sci. Pollut. Res..

[14] Cilia G., Resci I., Scarpellini R., Zavatta L., Albertazzi S., Bortolotti L., Nanetti A., Piva S. (2023). Antimicrobial-Resistant Environmental Bacteria Isolated Using a Network of Honey Bee Colonies (*Apis mellifera* L. 1758). Transbound. Emerg. Dis..

[15] Piva S., Giacometti F., Marti E., Massella E., Cabbri R., Galuppi R., Serraino A. (2019). Could honey bees signal the spread of antimicrobial resistance in the environment?. Lett. Appl. Microbiol..

[16] Resci I., Zavatta L., Piva S., Mondo E., Albertazzi S., Nanetti A., Bortolotti L., Cilia G. (2024). Predictive statistical models for monitoring antimicrobial resistance spread in the environment using *Apis mellifera* (L. 1758) colonies. Environ. Res..

[17] Saccà M.L., Resci I., Cilia G. (2024). Phenotypic and genotypic antimicrobial resistance patterns in honey bee (*Apis mellifera* L.) bacterial symbionts. Environmental Science and Pollution Research.

[18] Resci I., Cilia G. (2023). The use of honey bee (*Apis mellifera* L.) as biological monitors for pathogenic bacteria and antimicrobial resistance: A systematic review. Environ. Pollut..

[19] Laconi A., Tolosi R., Mughini-Gras L., Mazzucato M., Ferrèd N., Carraroa L., Cardazzo B., Capolongo F., Merlanti R., Piccirillo A. (2022). Beehive products as bioindicators of antimicrobial resistance contamination in the environment. Sci. Total Environ..

[20] Raymann K., Moran N.A. (2018). The role of the gut microbiome in health and disease of adult honey bee workers. Curr. Opin. Insect Sci..

[21] Kwong W.K., Moran N.A. (2016). Gut microbial communities of social bees. Nat. Rev. Microbiol..

[22] Sun H., Mu X., Zhang K., Lang H., Su Q., Li X., Zhou X., Zhang X., Zheng H. (2022). Geographical resistome profiling in the honeybee microbiome reveals resistance gene transfer conferred by mobilizable plasmids. Microbiome.

[23] Zheng H., Steele M.I., Leonard S.P., Motta E.V.S., Moran N.A. (2018). Honey bees as models for gut microbiota research. Lab Anim..

[24] Kešnerová L., Emery O., Troilo M., Liberti J., Erkosar B., Engel E. (2020). Gut microbiota structure differs between honeybees in winter and summer. ISME J..

[25] Baffoni L., Alberoni D., Gaggia F., Braglia C., Stanton C., Ross P.R., Di Gioia D. (2021). Honeybee Exposure to Veterinary Drugs: How Is the Gut Microbiota Affected?. Microbiol. Spectr..

[26] Li J.H., Evans J.D., Li W.F., Zhao Y.Z., DeGrandi-Hoffman G., Huang S.K., Li Z.G., Hamilton M., Chen Y.P. (2017). New evidence showing that the destruction of gut bacteria by antibiotic treatment could increase the honey bee’s vulnerability to Nosema infection. PLoS ONE.

[27] Liu Y., Jia S., Wu Y., Zhou N., Xie Y., Wei R., Huang Z., Chen Y., Hu F., Zheng H. (2024). Tetracycline-induced gut community dysbiosis and Israeli Acute Paralysis Virus infection synergistically negatively affect honeybees. Ecotoxicol. Environ. Saf..

[28] Mallory E., Freeze G., Daisley B.A., Allen-Vercoe E. (2024). Revisiting the role of pathogen diversity and microbial interactions in honeybee susceptibility and treatment of *Melissococcus plutonius* infection. Front. Vet. Sci..

[29] Raymann K., Shaffer Z., Moran N.A. (2017). Antibiotic exposure perturbs the gut microbiota and elevates mortality in honeybees. PLoS Biol..

[30] Tian B., Fadhil N.H., Powell J.E., Kwong W.K., Moran N.A. (2012). Long-term exposure to antibiotics has caused accumulation of resistance genes in the honeybee gut microbiota. mBio.

[31] Mulchandani R., Wang Y., Gilbert M., Van Boeckel T.P. (2023). Global trends in antimicrobial use in food-producing animals: 2020 to 2030. PLoS Global Public Health.

[32] Tomassone L., Scali F., Formenti N., Alborali G.L., Aragrande M., Canali M., Romanelli C., Suprani V., De Meneghi D. (2024). Evaluation of ‘ClassyFarm’, the Italian integrated surveillance system of livestock farms, in the context of antimicrobial use and antimicrobial resistance. Ital. J. Anim. Sci..

[33] DG Health and Food Safety (2022). Report on the Implementation of the Farm to Fork Strategy.

[34] Classyfarm. https://www.classyfarm.it/index.php/en/.

[35] Neumann K., Elbersen B.S., Verburg P.H. (2009). Modelling the spatial distribution of livestock in Europe. Landsc. Ecol..

[36] WHOA, List of Antimicrobial Agents of Veterinary Importance. https://www.woah.org/app/uploads/2021/06/202501-en-woah-trd-list.pdf.

[37] Nagano N., Shibata N., Saitou Y., Nagano Y., Arakawa Y. (2003). Nosocomial Outbreak of Infections by *Proteus mirabilis* That Produces Extended-Spectrum CTX-M-2 Type–Lactamase. J. Clin. Microbiol..

[38] Ng L.K., Martin I., Alfa M., Mulvey M. (2001). Multiplex PCR for the detection of tetracycline resistant genes. Mol. Cell. Probes.

[39] Gay K., Robicsek A., Strahilevitz J., Park C.H., Jacoby G., Barrett T.J., Medalla F., Chiller T.M., Hooper D.C. (2006). Plasmid-mediated quinolone resistance in non-Typhi serotypes of *Salmonella enterica*. Clin. Infect. Dis..

[40] Klindworth A., Pruesse E., Schweer T., Peplies J., Quast C., Horn M., Glo¨ckner F.O. (2013). Evaluation of general 16S ribosomal RNA gene PCR primers for classical and next-generation sequencing-based diversity studies. Nucleic Acids Res..

[41] Apprill A., McNally S., Parsons R., Weber L. (2015). Minor revision to V4 region SSU rRNA 806R gene primer greatly increases detection of SAR11 bacterioplankton. Aquat. Microb. Ecol..

[42] Waseem H., Williams M.R., Stedtfeld R.D., Hashsham S.A. (2017). Antimicrobial Resistance in the Environment. Water Environ. Res..

[43] Rogowska J., Gałęzowska G., Zimmermann A. (2024). Challenges and Current Trends in Preventing Antimicrobial Resistance in EU Water Law Context. Antibiotics.

[44] Kelbrick M., Hesse E., O’ Brien S. (2023). Cultivating antimicrobial resistance: How intensive agriculture ploughs the way for antibiotic resistance. Microbiology.

[45] Zhang Y., Su J.Q., Liao H., Breed M.F., Yao H., Shangguan H., Li H.Z., Sun X., Zhu Y.G. (2023). Increasing Antimicrobial Resistance and Potential Human Bacterial Pathogens in an Invasive Land Snail Driven by Urbanization. Environ. Sci. Technol..

[46] Agarwal V., Yue Y., Zhang X., Feng X., Tao Y., Wang J. (2023). Spatial and temporal distribution of endotoxins, antibiotic resistance genes and mobile genetic elements in the air of a dairy farm in Germany. Environ. Pollut..

[47] Azaglo G.S.K., Khogali M., Hann K., Pwamang J.A., Appoh E., Appah-Sampong E., Agyarkwa M.A., Fiati C., Kudjawu J., Hedidor G.K. (2021). Bacteria and Their Antibiotic Resistance Profiles in Ambient Air in Accra, Ghana, February 2020: A Cross-Sectional Study. Trop. Med. Infect. Dis..

[48] de Rooij M.M.T., Hoek G., Schmitt H., Janse I., Swart A., Maassen C.B.M., Schalk M., Heederik D.J.J., Wouters I.M. (2019). Insights into Livestock-Related Microbial Concentrations in Air at Residential Level in a Livestock Dense Area. Environ. Sci. Technol..

[49] Martinez J.L. (2009). Environmental pollution by antibiotics and by antibiotic resistance determinants. Environ. Pollut..

[50] Van Goethem M.W., Pierneef R., Bezuidt O.K.I., Van De Peer Y., Cowan D.A., Makhalanyane T.P. (2018). A reservoir of ‘historical’ antibiotic resistance genes in remote pristine Antarctic soils. Microbiome.

[51] Wellington E.M.H., Boxall A.B.A., Cross P., Feil E.J., Gaze W.H., Hawkey P.M., Johnson-Rollings A.S., Jones D.L., Lee N.M., Otten W. (2013). The role of the natural environment in the emergence of antibiotic resistance in Gram-negative bacteria. Lancet Infect. Dis..

[52] Engel P., Kwong W.K., McFrederick Q., Anderson K.E., Barribeau S.M., Chandler J.A., Cornman R.S., Dainat J., de Miranda J.R., Doublet V. (2016). The Bee Microbiome: Impact on Bee Health and Model for Evolution and Ecology of Host-Microbe Interactions. mBio.

[53] Jones J.C., Fruciano C., Hildebrand F., Al Toufalilia H., Balfour N.J., Bork P., Engel P., Ratnieks F.L., Hughes W.O. (2018). Gut microbiota composition is associated with environmental landscape in honey bees. Ecol. Evol..

[54] Muñoz-Colmenero M., Baroja-Careaga I., Kovačić M., Filipi J., Puškadija Z., Kezić N., Estonba A., Büchler R., Zarraonaindia I. (2020). Differences in honey bee bacterial diversity and composition in agricultural and pristine environments—A field study. Apidologie.

[55] Ludvigsen J., Porcellato D., L’Abée-Lund T.M., Amdam G.V., Rudi K. (2017). Geographically widespread honeybee-gut symbiont subgroups show locally distinct antibiotic-resistant patterns. Mol. Ecol..

[56] Italian Ministry of Health (2025). European Sales and Use (ESUAvet) Annual Surveillance.

[57] Jin M.J., Barron A.B., He S.Y., He X.J., Huang Q., Zhang L.Z., Wang Z.L., Wu X.B., Yan W.Y., Zeng Z.J. (2025). Bombella intestini: A probiotic honeybee (*Apis mellifera*) gut bacterium. J. Insect Physiol..

[58] Steele M.I., Motta E.V.S., Gattu T., Martinez D., Moran N.A. (2021). The gut microbiota protects bees from invasion by a bacterial pathogen. Microbiol. Spectr..

